# Honest Errors and Self-Retraction should Not be Stigmatized or Penalized

**DOI:** 10.12669/pjms.341.14470

**Published:** 2018

**Authors:** Shaukat Ali Jawaid

Faculty members, research scientists and academicians are currently under lot of pressure to publish their work for various reasons but it has also created lot of problems not only for the authors but also for the medical journal editors. Every author wish to see his/her work in print immediately but it is not possible for the editors to oblige them as formatting, plagiarism check, internal and external review, revision of the manuscript by authors, post acceptance editing and final publication all take time with which a vast majority of the authors are not familiar.^1^ Even otherwise, medical scientific writing has evolved through different phases though the revolution in information technology has accelerated the pace of development. However, to keep pace with the latest developments it not only requires a total commitment and devotion but also require adequate funding which may not be available with every one depending on the resources of the publishers and the business model which every journal has adopted.

Authors and editors both had to face certain hurdles, cross different barriers during their journey. Whenever some problem was identified, a solution was also found. In the past good old days, only correspondence author was required to give a written undertaking that the manuscript being submitted to a particular journal was an unpublished material and was exclusively being submitted to that particular journal which was accepted. All this was based on Trust but then this trust was shattered when different authors in the same manuscript started submitting the same manuscript to various journals in order to ensure early publication. Hence as soon as it was accepted by one journal, the authors would ask for withdrawing their manuscript from other journals which resulted in wasting lot of precious time of the editors and reviewers. So the solution was found and now all the listed authors have to give a written undertaking about its exclusive submission.

Similarly in the past, head of the department with keen interest in research would select a topic and ask different people working in the unit to work on that particular project. At the same time the staff in the unit will be working on more than one project/study. In a particular study if one was entrusted to do literature search, other will do the data analysis and interpretation while another one will prepare the draft which will then be looked at and approved by all the listed authors before it was submitted for publication to a journal. Here again it was trust between the different team members working in a unit. Since in the past no software was available to check for plagiarism, the mutual trust of the team members had assumed lot more importance and it was expected that each one will do his/her job honestly. Later on when software for plagiarism checking became available screening for plagiarism and detection of plagiarism became much easier. However, though different plagiarism checking software are available free on the net, they are not so effective and one has to subscribe Turnitin/iThenticate which is of course expensive and small journal with resource constraint could not afford it. Hence, if the reviewer had the facility of plagiarism check, they used it and the journal editors relied on the Reviewers report.

Plagiarism check and detection of scientific misconduct had become quite popular but neither all authors nor all the editors had the luxury of having access to this plagiarism check software like Turnitin/iThenticate till the Higher Education Commission, Government of Pakistan provided this facility to public sector medical and other teaching institutions including the medical universities. When this facility became available, some of the old published manuscripts were also scrutinized by the conscious authors and some of them did find that some of their colleagues had not been so honest and while working on a manuscript, they did plagiarized some portion in their write-up. Hence, when it was realized, detected, they opted for the right decision to request the editor to retract their paper which they did. Such admission of honest errors and self retraction should not be stigmatized or penalized by the authorities. The objective is to avoid any scientific misconduct and instead of taking any punitive measures, in such cases of self retraction, it will be much better to create awareness about plagiarism and upholding professional ethics. Moreover, if such things have taken place at a time when these facilities were not easily available it also calls for showing understanding. Now even if the authors have indulged in some scientific misconduct of plagiarism, the journal editors are able to detect it before it is published but in the past they too were handicapped due to lack of these facilities. Now it is extremely difficult for authors to get any such manuscript published in a good quality peer reviewed journal as all of them have plagiarism check facilities which they have themselves subscribed or have been provided by the HEC or the Reviewers utilize this facility to which they have access as faculty member of some institution which has been provided this facility by HEC.

At present following are some of the freely as well as commercially available online plagiarism detection tools which can be used for screening manuscripts for plagiarism.^2^

1. Cross Check™
2. http://www.ithenticate.com
3. https://turnitin.com/static/index
4. Viper (http://www.scanmyessay.com/plagiarism - free software)
5. Software like eTBLAST
6. SafeAssign™
7. WCopyFind™
8. http://www.checkforplagiarism.net
9. http://www.grammarly.com
10. Sometimes simple Google Search also helps in detecting plagiarism.

Ensuring integrity of research in multi-author studies was yet another problem. When any such case went to the courts, different authors used to put blame on others saying that he or she was responsible for this. Hence, International Committee of Medical Journal Editors (ICMJE) after thorough discussion came up with an additional criteria No. 4 in its authorship Guidelines which states that:^3^

“Agreement to be accountable for all aspects of the work in ensuring that questions related to the accuracy or integrity of any part of the work are appropriately investigated and resolved.”

Most of the journals now also ask the authors to give details of individual contribution of all the listed authors at the end of the manuscript so that in case of any reported scientific misconduct later, the concerned author is penalized instead of penalizing all the listed authors.

In view of the above, we have to look at all these issues in their correct perspective while taking any decision. Punitive measures do not work all the time and in certain genuine cases, restraint, warning and forgiveness works better. Just like there is a difference between “Medical Errors” and “Professional negligence”, there is a difference in “Honest Errors” as well as “Self-Retraction” and deliberate plagiarism wherein the authors accept their mistakes hence they should be handled differently. There are examples where the scientific community has rewarded scientists who retract papers for honest errors. Retraction Watch is doing the right thing in this regard. It will be much better if all the stake holders promote research integrity and highlight examples of scientists who benefitted professionally by doing the right things. Reforms in Retraction are also needed to distinguish between honest errors, self retraction and deliberate scientific misconduct. At present most of the researchers and the journal editors as well as institutions and regulatory bodies have poor understanding about these things. Many such problems reported are the result of Lack of knowledge and experience. In addition there are limitations in journal policies and most of them are not equipped to handle cases of self- retraction efficiently.^4^
